# Construction of Effective Nanosensor by Combining Semiconducting Polymer Dots with Diphenylcarbazide for Specific Recognition of Trace Cr (VI) Ion in Water and Vitro

**DOI:** 10.3390/nano12152663

**Published:** 2022-08-03

**Authors:** Xilin Dou, Quan Wang, Tao Zhu, Zhaoyang Ding, Jing Xie

**Affiliations:** College of Food Science and Technology, Shanghai Ocean University, Shanghai 201306, China; m200300830@st.shou.edu.cn (X.D.); 13817695068@163.com (Q.W.); taozhu0823@163.com (T.Z.)

**Keywords:** hexavalent chromium, fluorescence sensing, Pdots, diphenylcarbazide

## Abstract

Hexavalent chromium (Cr (VI)) ion, as highly toxic environmental pollution, severely endangers the ecological environment and public health. Herein, a fluorescent nanosensor (PFO-DPC) was constructed by combining semiconducting polymer dots with diphenylcarbazide (DPC) for sensing Cr (VI) ion in aqueous solution and living cells. DPC and poly (styrene-co-maleic anhydride) (PSMA) polymer mixed with polyfluorene (PFO) were utilized for selectively indicating Cr (VI) ion and improving the efficiency of detection, respectively. The presence of Cr (VI) ion effectively turned off the blue and green fluorescence of PFO-DPC in the aqueous environment, and the fluorescence quenching efficiency exhibited a good linear relationship between the range of 0.0 to 2.31 nM (R^2^ = 0.983) with a limit of detection (LOD) of 0.16 nM. The mechanism of fluorescence quenching could possibly be attributed to the internal filtration effect (IFE). Additionally, PFO-DPC showed a satisfactory performance in monitoring intracellular Cr (VI) ion. Our results indicate that the sensor is promising in various applications.

## 1. Introduction

Hexavalent chromium (Cr (VI)) ion is one of the toxic heavy metal ions and is usually produced as a pollutant in various industries including tanning, electroplating, mining, etc. [1] As it enters nature environments, Cr (VI) ion intends to remain, migrate, and accumulate in aquatics environments or soil and would be hardly biodegraded, resulting in a negative effect on ecological security and public health [2]. Moreover, the ingestion of Cr (VI) ion may cause some severe diseases such as chronic ulcers, diarrhea and renal failure [3]. Therefore, the United States Environmental Protection Agency (US EPA) and World Health Organization (WHO) recommend that the concentration of Cr (VI) ion in drinking water should be less than 0.1 µg/mL and 16 µg/L, respectively [4,5]. In recent years, various methods were developed to detect Cr (VI) ion, such as plasma mass spectrometry (ICP-MS) [6], X-ray fluorescence [7], voltammetry [8], immunoassay-based detection [9], etc. However, these methods required complex pretreatment procedures, expensive and complicated equipment, time-consumption detecting processes or highly trained operators. Hence, it is a very significant research topic to design efficient, rapid, facile and sensitive assay methods to detect Cr (VI) ion.

Fluorescence assay has attracted much attention due to its convenience, rapid detection process and relative cost-effectiveness [10,11]. Some reports have focused on the fluorescent methods of Cr (VI) ion detection using different fluorescent materials such as carbon dots [12,13], metal organic frameworks [14], organic complexes [15] or boron carbon oxynitride [16]. However, there are a few reports about the detection of Cr (VI) ion using semiconducting polymer dots (Pdots) as fluorescent probes. Pdots exhibit high absorption cross sections, good photostability and particularly fluorescence brightness that can be magnitudes higher than that of organic dyes and tens of times greater than that of quantum dots [17,18]. Thus, fluorescent sensors based on Pdots have been greatly designed and prepared [19,20]. Moreover, the low toxicity and remarkable biological compatibility of Pdots have attracted researchers to utilize them in biomedical fields such as drug delivery [21,22,23], bioimaging [24,25] and biosensing [26,27]. Diphenylcarbazide (DPC) is frequently reported as a Cr (VI) ion indicator, which is based on the reduction of Cr (VI) ion to Cr (III) ion while DPC is oxidized to diphenylcarbazone (DPCA) at the same time, and then Cr (III) ion and DPCA would form a purple-colored complex. The typical DPC method is the colorimetric method [28,29], and the combination of DPC reagent and the other method has been also explored. Recently, Zhi et al. reported a Rayleigh scattering spectral probe consisting of DPC and liquid crystal trans for Cr (VI) ion detection in aqueous solution [30]. However, to our knowledge, the DPC reagent has been barely utilized to construct fluorescent probes for sensing Cr (VI) ion.

**Scheme 1:**
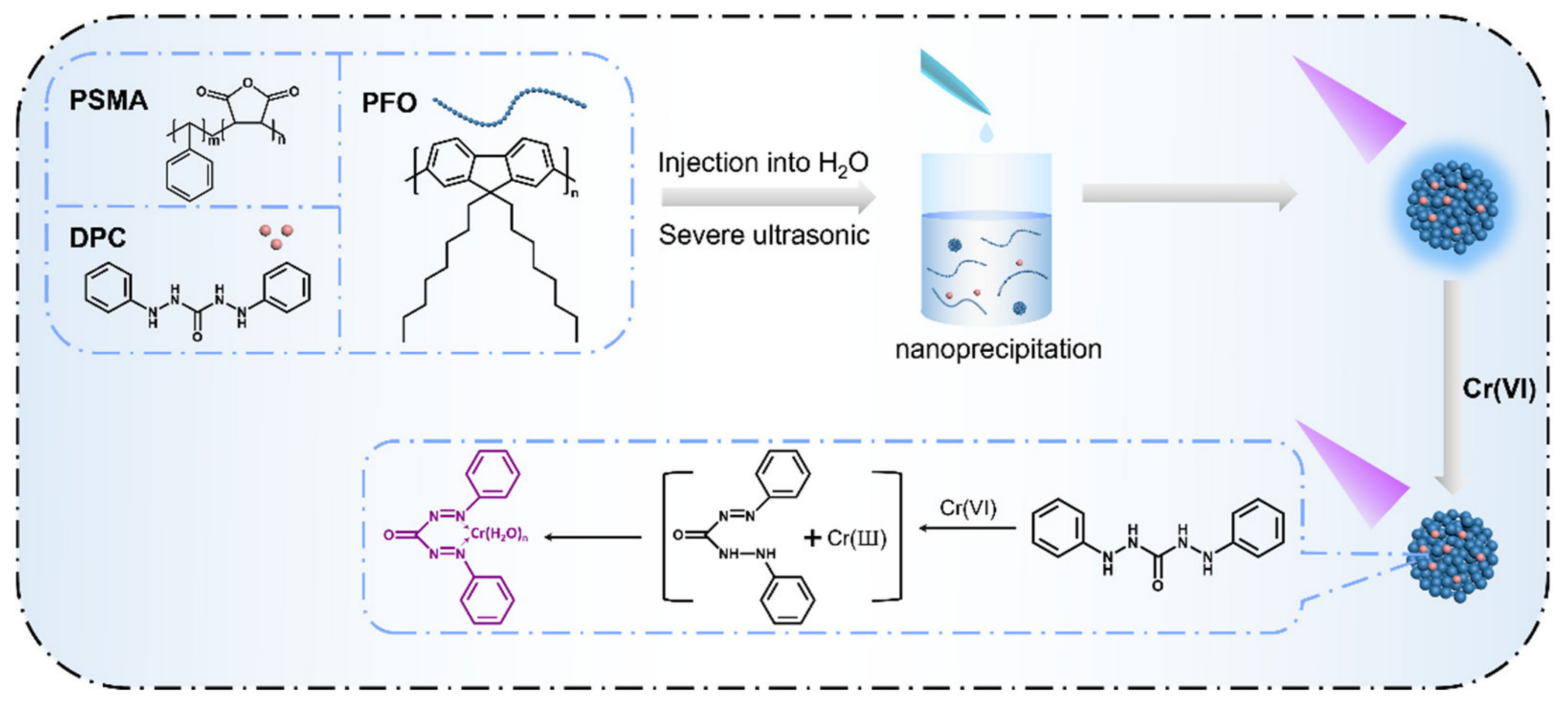
Schematic diagram of preparation of PFO-DPC and fluorescent detection for Cr (VI) ion with PFO-DPC.

In this work, a fluorescent nanosensor PFO-DPC based on highly bright Pdots was reported for the detection of Cr (VI) ion. As shown in Scheme 1, PFO-DPC was synthesized using poly (9,9-dioctylfluorene-2,7-diyl) (PFO) polymer, copolymer poly (styrene-co-maleic anhydride) (PSMA) and diphenylcarbazide (DPC) molecules. PFO is an excellent light-emitting polymer due to its highly efficient photoluminescence, great solubility, good chemical and thermal stability and tunable properties through copolymerization [31,32]. PSMA was utilized to generate carboxyl groups on the PFO-DPC surface, which could improve the water solubility of PFO-DPC and capture metal ions through chelation [33]. Furthermore, DPC dopped in the Pdots could specifically recognize Cr (VI) ion, in which Cr (VI) ion selectively bound to DPC to form a purple complex and turned off the bright fluorescence of PFO-DPC, which may be subject to the internal filter effect (IFE). The developed PFO-DPC offered the merits of sensitivity, selectivity, visualization and stability. Moreover, PFO-DPC also showed good performance in intracellular Cr (VI) ion detection, substantially expanding the application value of the sensor.

## 2. Materials and Methods

### 2.1. Materials and Apparatus

Poly (9,9-dioctylfluorene-2,7-diyl) (PFO) and poly (styrene-co-maleic anhydride) (PSMA) were purchased from Sigma-Aldrich. Tetrahydrofuran (THF; anhydrous, ≥99.9%, inhibitor-free), diphenylcarbazide (DPC), K_2_Cr_2_O_7_, NaCl, MgCl_2_, KCl, CaCl_2_, FeCl_3_ 6H_2_O, CuCl_2_ 2H_2_O, NiCl_2_, KMnO_4_, acetic acid, boric acid, phosphoric acid, HCl and NaOH were obtained from Sinopharm Chemical Reagent Co., Ltd. (Shangai, China).

The apparatus used in the work included a transmission electron microscope (Talos F200X G2, Thermo Fisher, Waltam, MA, USA); dynamic light scattering system (DLS, Malvern Zetasizer Nano ZS, Malvern, United Kingdom); U-3900 ultraviolet–visible (UV–vis) spectrophotometer (Hitachi, Japan); F-7000 fluorescence spectrophotometer (Hitachi, Japan) and Laser Scanning Confocal Microscopy (Carl Zeiss, Germany).

### 2.2. Synthesis of PFO-DPC

PFO-DPC was synthesized by the nanoprecipitation method. The polymers PFO and PSMA were dissolved in anhydrous THF with a PFO concentration of 1 mg/mL and PSMA concentration of 1 mg/mL. Then, 0.5 mL PFO solution, 0.5 mL PSMA solution, 0.5 mL DPC solution (4.1 mM) and 3.5 mL anhydrous THF were mixed, and the mixture was homogenized by sonication. Next, 5 mL of the resulting homogeneous solution was quickly injected into 10 mL of cold ultrapure water under high sonication power. Finally, the THF in the solution mixture was removed by bubbling nitrogen on a hot plate at 100 °C for 60 min followed by filtration through a 0.22 μm syringe filter to remove aggregates.

### 2.3. Fluorescence Spectra Detection Experiments

The fluorescent spectra of PFO-DPC in different pHs were obtained in Britton–Robinson buffer (BR) buffer (0.04 M phosphoric acid, 0.04 M boric acid and 0.04 M acetic acid) and pH values were adjusted by using NaOH and HCl. The detection of Cr (VI) ion was performed in BR buffer solution (pH = 7.0). A total of 100 μL of PFO-DPC (50 µg/mL) solutions and 900 μL Cr (VI) ion samples with different concentrations were added into 5 mL polypropylene centrifuge tubes. After mixing for 15 min, their fluorescence spectra were acquired at an excitation wavelength of 380 nm. The selectivity of PFO-DPC toward Cr (VI) ion was investigated by adding other metal ions (Na^+^, Mg^2+^, K^+^, Ca^2+^, Fe^3+^, Cu^2+^, Ni^2+^ and MnO_4_^-^) at 5.0 nM into the PFO-DPC solution in the presence and absence of Cr (VI) ion, and then fluorescence spectra were obtained as described above. Fluorescent stability was evaluated by recording the change in fluorescence intensity PFO-DPC within two weeks.

### 2.4. Cell Culture and Imaging

HeLa cells were used for fluorescent imaging and the HeLa cells were provided by Nantong University. The HeLa cells were incubated in RPMI-1640 medium supplemented with 1% penicillin–streptomycin solution, 10% fetal bovine serum (FBS) at 37 °C in a 5:95 CO_2_-air incubator (100% humidity). The cells were cultured for three days and then incubated with PFO-DPC (5 µg/mL) for 60 min at 37 °C in an incubator followed by washing with PBS three times and bathing in PBS buffer. Next, the cells were further treated with Cr (VI) ion at different concentrations for 15 min at 37 °C. After removing the culture medium and washing with PBS several times, the confocal fluorescence images of HeLa cells were taken by Carl Zeiss Confocal LSM710 at room temperature.

## 3. Results and Discussion

### 3.1. Characterization of PFO-DPC

PFO-DPC consisted of PFO polymer, copolymer PSMA and DPC molecules. The doped DPC, as the specifical indicator of Cr (VI) ion, could form a purple complex in the presence of Cr (VI) ion. The incorporation of PSMA benefited the sensing efficiency by enhancing the water solubility of PFO-DPC, improving contact between the sensor and metal ions and capturing Cr (VI) ion through chelation [34].

**Figure 1:**
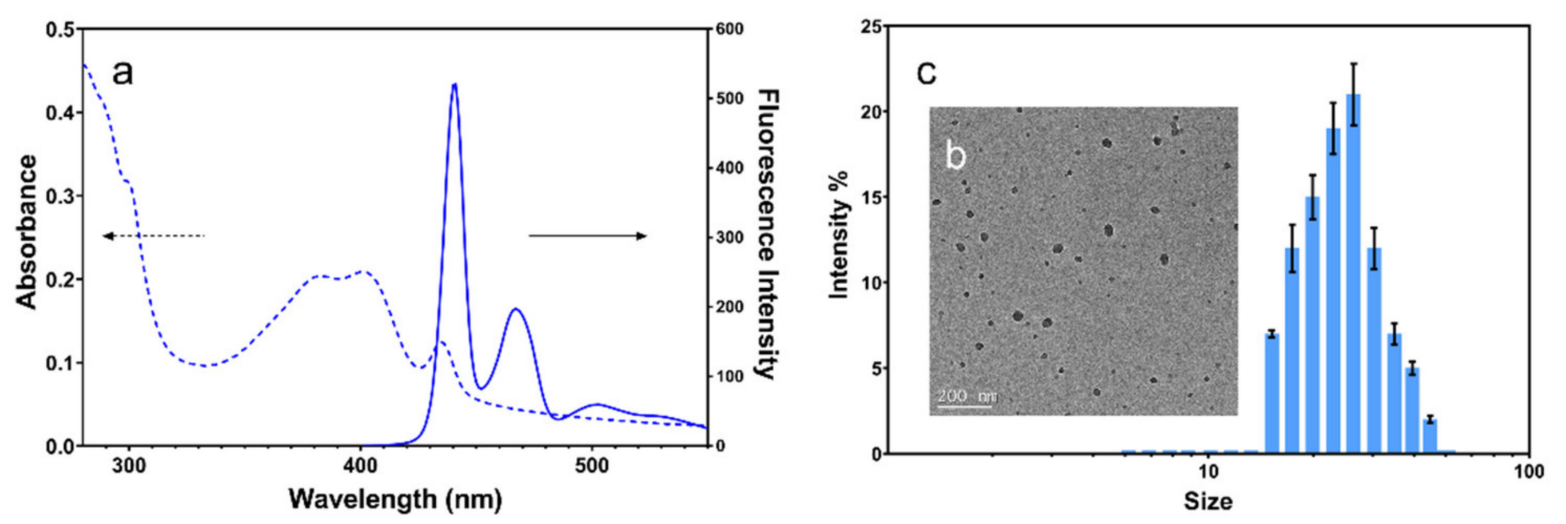
Fluorescence spectra and absorption spectra: (**a**) TEM image (**b**) and particle size distribution diagram (**c**) of PFO-DPC.

As shown in Figure 1a, PFO-DPC exhibited intensive absorption in the region of 330–440 nm. Slightly different from PFO polymer diluted in organic solvent like tetrahydrofuran (THF), PFO-DPC had an extra absorption band at 430 nm assigned to the β phase of PFO polymer. The α phase of PFO is disordered and randomly oriented, whereas the β phase is ordered and flat in conformation [32,35]. The formation of the β phase of PFO reflected the compact structure of PFO-DPC. The TEM image (Figure 1b) shows that PFO-DPC was monodispersed and nearly spherical. Moreover, according to the DLS analysis (Figure 1c), PFO-DPC exhibited a polydispersity index (PDI) of 0.178 with a mean diameter of 27 nm, indicating the small size and narrow monodispersed particle size distribution of the sensor. The characteristics of monodispersedness and small size endowed PFO-DPC with good water solubility and potential application in the biosensing field.

### 3.2. Sensitivity of Cr (VI) Ion Detection

**Figure 2:**
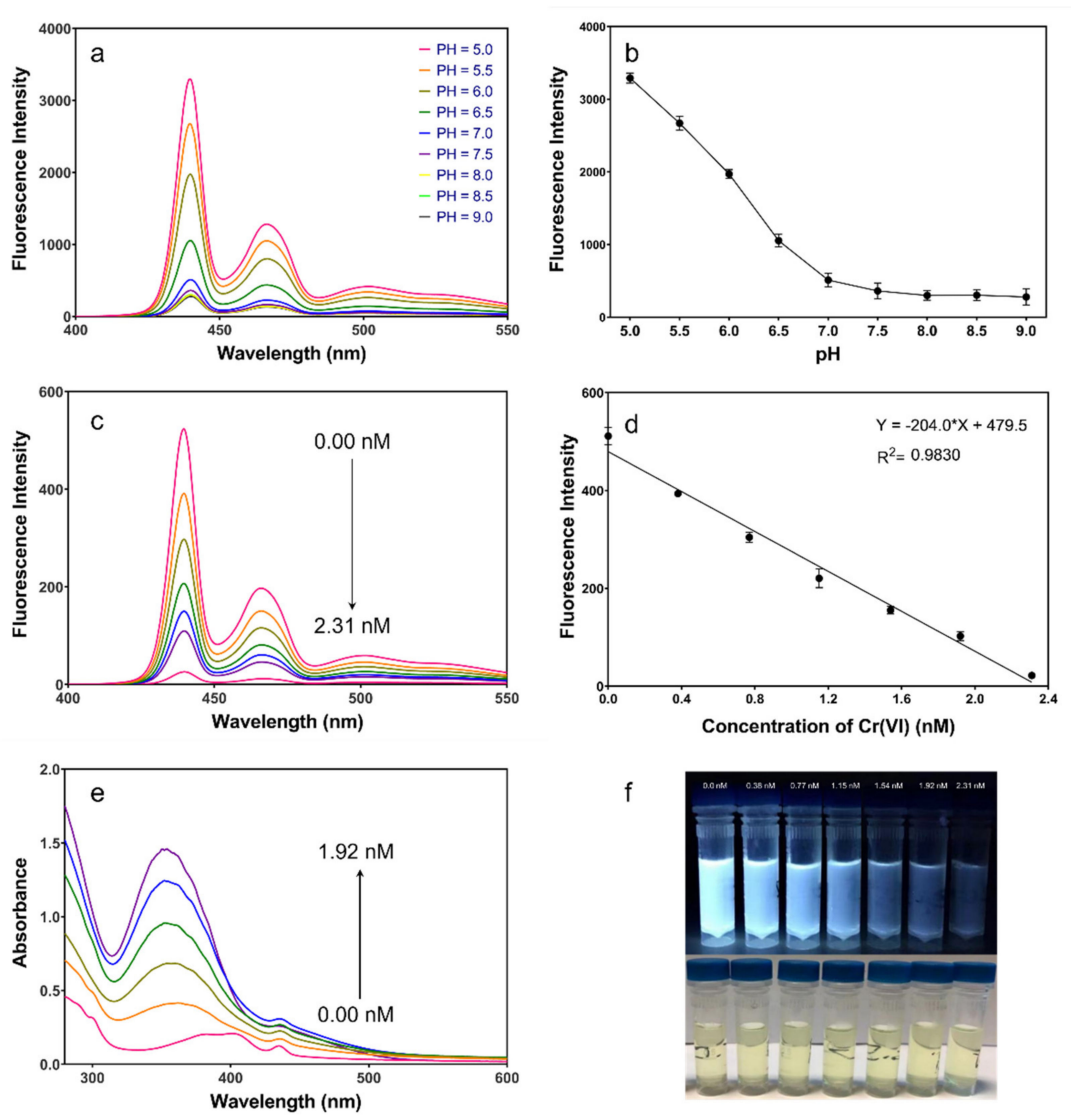
(**a**) Fluorescence spectra of PFO-DPC in different pH. (**b**) Effect of pH on the fluorescence intensity of PFO-DPC. (**c**) Fluorescence spectra of PFO-DPC at various Cr (VI) ion concentrations (0–2.31 nM). (**d**) Linear relationship between fluorescence intensity of PFO-DPC (emission 440 nm) and Cr (VI) ion concentrations. (**e**) UV–vis absorption spectra of PFO-DPC in different concentrations of Cr (VI) ion. (**f**) Naked eye photograph of PFO-DPC under the UV light (top) and visible light (bottom) with various concentrations of Cr (VI) ion.

The effect of pH on fluorescence intensity and quenching efficiency was investigated by exploring PFO-DPC at different pH values. In the pH range 5.0–9.0, the fluorescence intensity first decreased (pH 5.0–7.0) and then kept stable (pH 7.0–9.0) (Figure 2a,b). Because the fluorescence intensity remained almost unchanged from pH 7.0 to pH 9.0, the solution at low pH might cause a repulsion effect between Cr (VI) ion and PFO-DPC. In addition, because the design of this fluorescent probe aimed to apply for determination of Cr (VI) ion in the water environment and even the physiological environment, pH 7.0 was selected for the fluorescence detection of Cr (VI) ion.

Figure 2c showed that the introduction of Cr (VI) ion could bring a remarkable fluorescence quenching of over 95% of the primaeval fluorescence intensity. Moreover, Figure 2d indicated a good linear relationship was obtained between the fluorescence intensity of PFO-DPC and Cr (VI) ion concentration in the range from 0.0 to 2.31 nM (y = −204.0x + 479.5, R^2^ = 0.983). Moreover, the low detection limit (LOD) was 0.16 nM. The quenching effect of PFO-DPC could be directly caught by the naked eye in Figure 2f. Under the UV light, the fluorescence intensity of PFO-DPC obviously decreased with the incremental concentration of Cr (VI) ion, while no obvious color change was observed under the visible light.

The possible mechanism of Cr (VI) ion detection was also discussed. Figure 2e showed that as the concentration of Cr (VI) ion increased, the UV–vis spectra of PFO-DPC increased in absorbance intensity and a broad band at 350 nm occurred, which is attributed to the absorption spectra of Cr (VI) ion [36]. No significant changes (red shift or blue shift) occurred in the absorption spectra of PFO-DPC after adding Cr (VI) ion at different concentrations, indicating no new substance was produced in the mixture of PFO-DPC and Cr (VI) ion. Thus, static quenching as well as photoinduced electron transfer (PET) were not responsible for the fluorescence quenching. As shown in Figure 1a, the absorption spectra of PFO-DPC exhibited three bands peaked at 380 nm, 400 nm and 440 nm. The incremental Cr (VI) ion concentration led to the broad band at 440 nm increasing in absorbance intensity (Figure 2e), which overlapped with the emission band of PFO-DPC. Furthermore, Cr (VI) ion could absorb the excitation light at 380 nm. Therefore, the fluorescence quenching was possibly ascribed to IFE.

### 3.3. Selectivity and Stability of PFO-DPC

**Figure 3:**
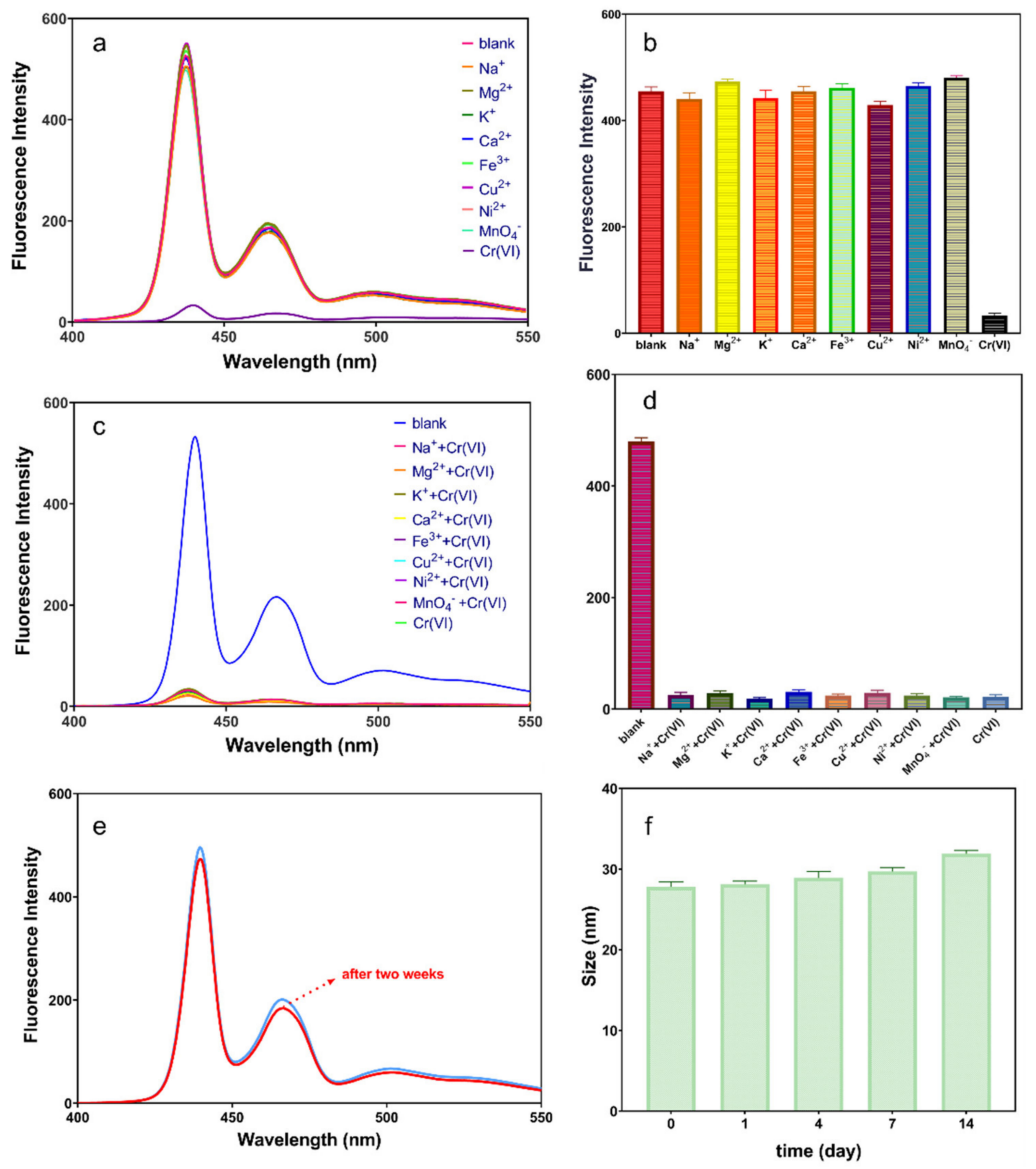
Fluorescence spectra of PFO-DPC with various metal ions in the absence (**a**) and presence (**c**) of Cr (VI) ion. Fluorescence response of PFO-DPC (emission 440 nm) with various metal ions in the absence (**b**) and presence (**d**) of Cr (VI) ion. The fluorescent stability of PFO-DPC (**e**). The structural stability of PFO-DPC (**f**).

Due to the specific reaction between Cr (VI) ion and DPC, a great selectivity of PFO-DPC was anticipated. To further evaluate the selectivity of PFO-DPC for Cr (VI) ion, the fluorescence responses to representative metal ions in aqueous solution (Na^+^, Mg^2+^, K^+^, Ca^2+^, Fe^3+^, Cu^2+^, Ni^2+^ and MnO_4_^−^) at 5.0 nM were examined. As shown in Figure 3a,b, only Cr (VI) ion (2.31 nM) effectively quenched the fluorescence of PFO-DPC, and the existence of other metal ions barely affected the quenching effect of Cr (VI) ion even at more than two-fold concentration of Cr (VI) ion (Figure 3c,d), indicating a high selectivity of the fluorescence sensor toward Cr (VI) ion.

The stability of fluorescence sensors is a significant factor to evaluate their practical value. Therefore, the initial and final fluorescence intensity and particle diameter of PFO-DPC within two weeks were recorded. As shown in Figure 3e, the fluorescence intensity of PFO-DPC only slightly decreased after two weeks of storage time. At the same time, PFO-DPC did not severely aggregate or decompose during the storage time (Figure 3f). These results proved the high stability of PFO-DPC.

### 3.4. Cell Imaging

**Figure 4:**
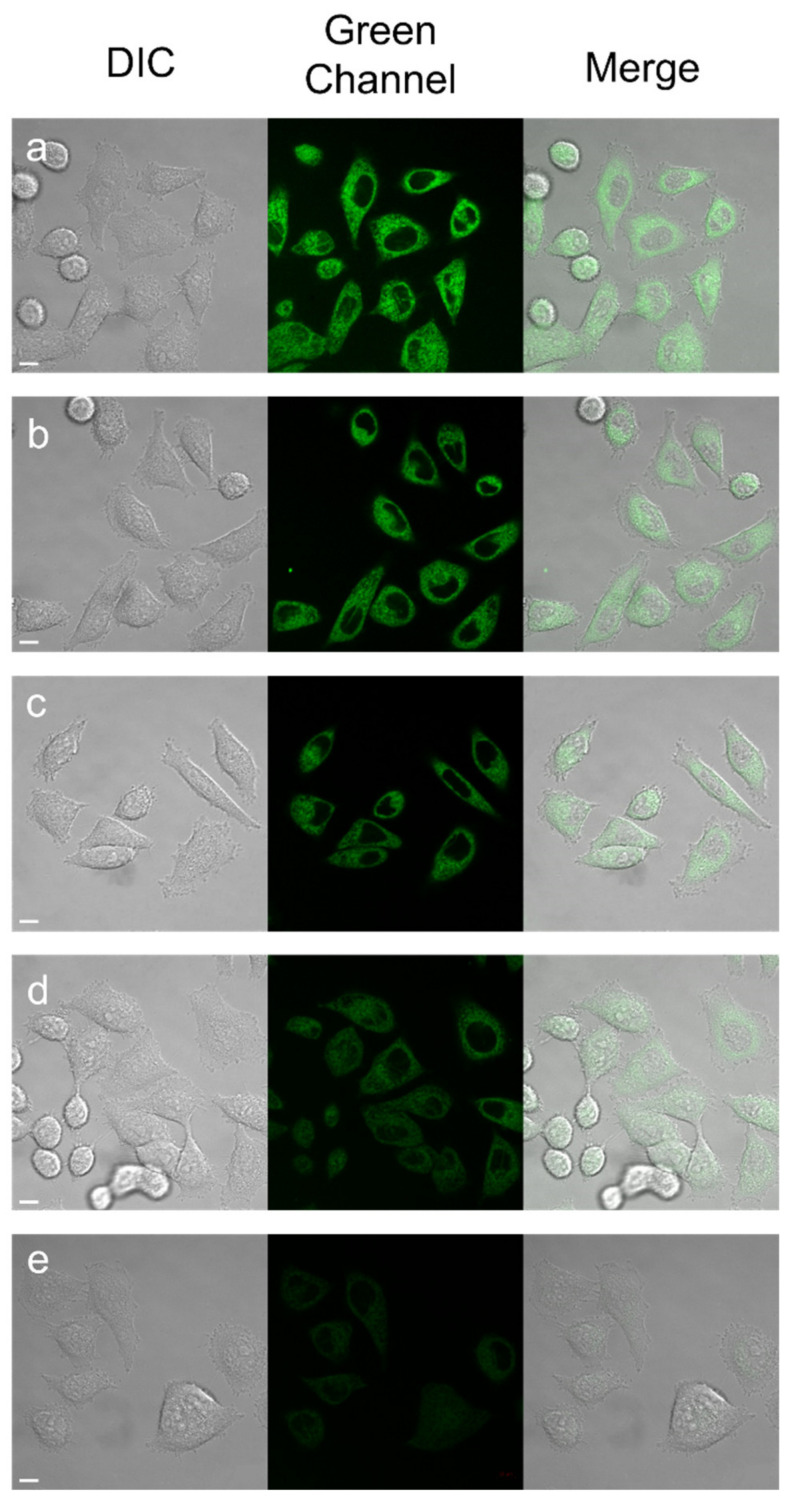
Confocal fluorescent images of HeLa cells after incubating with PFO-DPC and continuous exposure of exogenous Cr (VI) ion source treatment (0.00 nM (**a**), 0.38 nM (**b**), 0.77 nM (**c**), 1.15 nM (**d**) and 1.54 nM (**e**)). Scare bar = 10 µm.

The properties of small size, high stability and great selectivity render PFO-DPC suitable for biological application. Therefore, the responses of PFO-DPC in living cells were investigated. HeLa cells were incubated with 5 µg/mL PFO-DPC in RPMI-1640 medium supplemented with 10% fetal bovine serum (FBS) and 1% penicillin–streptomycin solution at 37 °C with 5% CO_2_ and 100% humidity. Then, the cells were washed with PBS buffer to remove the remaining PFO-DPC. The confocal fluorescent images were obtained in green channel. As shown in Figure 4a, a robust fluorescence signal caused by PFO-DPC could be observed in the intracellular compartments of living cells, indicating that the fluorescent sensor was greatly cell-permeable and kept the PL characteristic even in an intracellular environment. Furthermore, upon the addition of incremental Cr (VI) ion concentration, the intracellular fluorescence intensity of PFO-DPC gradually weakened and almost completely quenched with 1.54 nM Cr (VI) ion (Figure 4). The results demonstrated that the fluorescence quenching effect of PFO-DPC internalized by living cells could effectively visualize and determine the intracellular Cr (VI) ion at various concentrations.

## 4. Conclusions

In summary, a fluorescence nanosensor PFO-DPC with DPC as a target indicator was developed for the sensitive and selective detection of Cr (VI) ion. PFO-DPC with good aqueous dispersity displayed a highly efficient fluorescence quenching effect toward to Cr (VI) ion in a water environment, and the IFE dominated in the possible detection mechanism. The high selectivity and stability properties of PFO-DPC enabled the sensor to be useful in different cases. In addition, PFO-DPC was practical for the intracellular assays. As evidenced by confocal imaging, PFO-DPC could detect Cr (VI) ion sensitively based on the quenching effect in living cells. Accordingly, we believe that PFO-DPC holds great promise for the detection of Cr (VI) ion in various fields.

## Data Availability

Not applicable.
